# Food Retailer Licensing: An Innovative Approach to Increasing Access to Healthful Foods

**DOI:** 10.5888/pcd9.120127

**Published:** 2012-11-29

**Authors:** Ian McLaughlin, Karen Kramer

**Affiliations:** Author Affiliation: Karen Kramer, ChangeLab Solutions, Oakland, California.

## Introduction

Imagine walking into your neighborhood food retailer and finding that the dairy section has no skim milk (although sodas are plentiful) and that the only fresh produce offered is potatoes and limes. This is the reality for many urban and rural low-income Americans, whose only nearby “grocery store” is a mini-mart or corner store that is primarily stocked with processed foods high in fat and sugar. More than 2 million people live in low-income rural areas where the nearest supermarket is more than 10 miles away, and 116 million Americans must travel more than a mile, often without a car, to find a supermarket (1,2).

Poor access to healthful foods and oversaturation of unhealthful foods correlates with overweight and obesity; therefore, increasing the availability of healthful foods at small food retailers is critical to improving the food environment in low-income areas and should be part of any comprehensive approach to reversing the obesity epidemic (3). Recognizing this, public health advocates have implemented programs to encourage small food retailers to sell more healthful foods, usually by offering incentives, technical assistance, and promotional materials (4).

Although these programs can be effective (5), they benefit only a small patchwork of low-income areas nationally, and because they are nonregulatory and labor- and resource-intensive, they have a limited reach, are vulnerable to funding cuts, and typically rely on incentives rather than enforceable standards.

ChangeLab Solutions has developed a Model Licensing Ordinance for Healthy Food Retailers (“Model Ordinance”) to provide a new policy option for improving access to healthful food in communities (Model Ordinance available at http://changelabsolutions.org/publications/HFR-licensing-ord). ChangeLab Solutions (formerly Public Health Law & Policy) is a national nonprofit organization devoted to creating innovative community-based laws and policies that improve public health. Inspired by the successful use of licensing laws to reduce tobacco sales to youth, the Model Ordinance is designed to complement existing programmatic work by providing a broader reach, enforceable standards, and a more financially secure foundation.

## Overview of Licensing

Many local governments possess what is commonly referred to as “police power,” which allows them to regulate private conduct to protect the public’s health and safety (6–10). Licensing is one such method of regulation. For example, many municipalities invoke their police power to require stores to obtain a business license to operate and condition the license on payment of a fee, compliance with standards that promote health or safety, or both. If the standards are not satisfied, the municipality can suspend, revoke, or choose not to renew the license (11).

Licensing has long been used to implement public health policies. For example, municipalities often use their licensing power to enforce food safety and hygiene standards for restaurants. Similarly, licensing power is typically invoked to bar or limit sales of items, such as firecrackers, alcohol, and tobacco, that are potentially harmful to the public’s health. Local licensing ordinances for tobacco retailers have been effective in enforcing statewide laws prohibiting the sale of tobacco products to minors (12,13). Licensing provides local jurisdictions with a well-established vehicle for implementing public health policies in the retail environment.

Most municipalities impose few requirements when granting business licenses to food retailers. A common licensing scheme may, for example, require a food retailer to pay a fee and provide information about the store as a condition of doing business in the jurisdiction. Existing license requirements, however, provide a foundation on which advocates and policy makers can build to improve access to more healthful foods, and municipalities can adopt a licensing ordinance that requires food retailers to offer a baseline level of healthful foods as a condition of their license. At least one city, Minneapolis, Minnesota, has already taken this approach by adopting an ordinance that requires grocery stores, as a condition of obtaining and maintaining their business license, to stock certain categories of healthful foods (14).

Although zoning regulations can also be a valuable tool for promoting public health, licensing laws have advantages. Zoning requirements generally apply only to new establishments (to avoid infringing vested rights enjoyed by existing property owners); licensing requirements, however, can usually apply to new and existing businesses (15,16). Therefore, licensing can be a more efficient way to create immediate changes in the retail environment. (A comparison of zoning and licensing is available at http://changelabsolutions.org/publications/zoning-and-licensing).

## The Model Ordinance

Similar to tobacco retailer licensing laws, the Model Ordinance imposes license conditions to achieve its policy objective, in this case, improving access to healthful foods throughout an entire jurisdiction. Although such an ordinance may be structured in many ways, our approach focuses on these critical issues: appropriate performance standards, compliance, and enforcement.

### Appropriate performance standards

The substantive requirements of the Model Ordinance result from extensive research, including input from 21 members of the national Healthy Corner Stores Network (www.healthycornerstores.org) who participated in a comprehensive survey. In essence, they require retailers to dedicate a percentage of their selling area (which varies on the basis of store size) to staple foods and fresh produce. This approach is designed to achieve several key objectives.

First, this approach establishes a “healthy food baseline” that every food retailer must meet, ensuring community-wide access to fresh produce and staples. Second, it gives retailers maximum flexibility to select and adjust their stock, as seasons, customer preferences, and market conditions change. To ensure that store owners do not abuse this discretion (for example, by using their percentage of produce-selling area to stock only limes), the Model Ordinance requires each jurisdiction to determine a minimum number of varieties of produce that must be stocked and dictates that “staple foods” include dairy, protein, and whole grains in equal amounts.

Third, because the percentage of a selling area can be readily quantified in square footage, the performance standards are straightforward, easy to understand, and objectively verifiable by inspectors. Finally, the Model Ordinance allows for variations in local conditions by allowing each jurisdiction to determine the applicable selling area percentage. Communities will need to give these percentages careful thought because setting the bar too low would undermine the ordinance, while setting it too high could trigger resistance from food retailers, leading to lower compliance rates and higher enforcement costs.

### Maximizing compliance

Many small food retailers may fear that staples and fresh produce will sell more slowly than junk food and will spoil on the shelves, thus jeopardizing their often already thin profit margins. To address this valid concern and thus reduce noncompliance, the Model Ordinance requires municipalities to provide small food retailers with the technical advice and education necessary to make a successful transition to a more healthful inventory. For example, given that demand may be low or uncertain initially, municipalities should help small food retailers develop a marketing plan to address issues such as product placement, customer preferences, pricing, advertising, and customer incentives.

Technical advice on finding a reliable distributor of healthful foods and proper handling of fresh produce is critical, because profitability depends on the ability to consistently offer quality produce at reasonable prices. Finally, education can help small food retailers access grants or loans to support store upgrades, obtain WIC (Special Supplemental Nutrition Program for Women, Infants, and Children) certification, which may increase demand for more healthful foods, and understand the purpose of the ordinance. Retailer concerns about profitability must be taken seriously and addressed through appropriate assistance, education, and outreach. Anecdotal evidence suggests, however, that with proper support, the shift can be made without compromising profits; in some cases, profits have increased (17,18).

### Enforcement

Although meaningful enforcement is essential to the success of a healthy food retailer licensing ordinance, it must be carefully calibrated. If violators are treated too punitively, businesses could be unnecessarily jeopardized, increasing the risk of a backlash. Therefore, policy makers should consider that the overall goal is to promote a more healthful food environment — not to penalize retailers by putting them out of business. Accordingly, the Model Ordinance emphasizes education and technical support for food retailers, and a failure to comply is met with progressive, measured steps aimed at remediation, with suspension or loss of license considered only as a remedy of last resort.

## Conclusion

The obesity crisis has increased the public’s awareness that many Americans do not have ready access to healthful foods in their neighborhood stores. Retailer licensing is a well-established, yet underused, regulatory power that can be used to implement new public health policies needed to combat the obesity epidemic. A healthy food retailer licensing ordinance can be a step toward ensuring that people have access to staples and fresh produce at their local food stores, whether these stores are grocery supermarkets or corner mini-marts. Additional resources are available at http://changelabsolutions.org/publications/healthy-corner-stores.
